# Transient Neurogenic Bowel Dysfunction in a Case of Cocaine-Induced Spinal Cord Infarction

**DOI:** 10.7759/cureus.23834

**Published:** 2022-04-05

**Authors:** Luis M Nieto, Sharon I Narvaez, Anantratn Asthana, Amir Mohammed, Jami Kinnucan

**Affiliations:** 1 Internal Medicine, Wellstar Atlanta Medical Center, Atlanta, USA; 2 Internal Medicine, Facultad de Medicina, Universidad de Guayaquil, Guayaquil, ECU; 3 Family Medicine, Wellstar Atlanta Medical Center, Atlanta, USA; 4 Gastroenterology and Hepatology, Mayo Clinic, Jacksonville, USA

**Keywords:** constipation, neurogenic bowel dysfunction, upper gastrointestinal bleeding, spinal cord infarction, cocaine

## Abstract

A 23-year-old male presented to the hospital with altered mental status (AMS) and hypoglycemia requiring admission to the ICU. He had improvement in AMS after administration of dextrose 50% and naloxone and endorsed the use of alcohol, cocaine, and marijuana that morning. It was confirmed with a positive urine toxicology screen for cocaine and tetrahydrocannabinol (THC). During this hospital admission, his physical examination was notable for paraplegia with no motor abilities from the T6 dermatome and below. Sensation was intact throughout all dermatomes but he was found to have urinary retention. Workup included an abnormal MRI showing T2 signal spanning from T2-T8, raising a high suspicion of a probable acute ischemic spinal cord infarction. Several hours after admission, the patient began to exhibit the first signs of abnormal bowel function and experienced one episode of hematemesis, prolonging his ICU stay.

## Introduction

Cocaine use can be associated with abnormalities of the central nervous system (CNS) including presentations of intracranial hemorrhage, cerebral vasculitis, and seizures. A rare CNS complication with high morbidity is acute spinal cord infarction (SCI) [1,2]. More common etiology for SCI includes a disease involving the aorta [3]; however other less common causes include systemic hypoperfusion, embolism from a cardiac origin, vasculitis, infection, hematology disorders, and cocaine use [4-6]. Qi et al. described the significant impact on quality of life due to bowel dysfunction secondary to a spinal cord injury, which is preceded only by the loss of motor function [7]. Neurogenic bowel dysfunction (NBD) symptoms are broad and may vary between patients with combinations of constipation, fecal incontinence, abdominal distention, and bloating. We describe a case of transient neurogenic bowel dysfunction secondary to cocaine-induced spinal cord infarction.

## Case presentation

**Figure 1:**
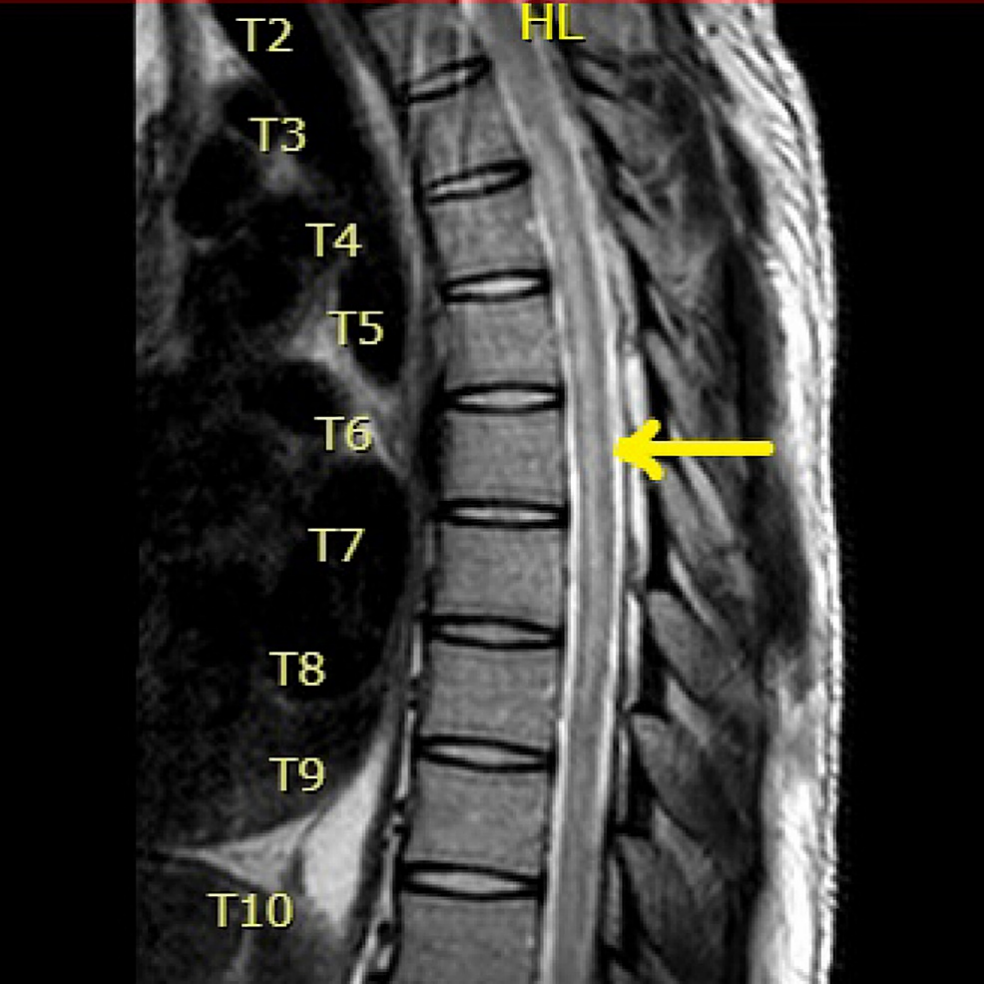
MRI of thoracic spine shows abnormal T2 signaling spanning from T2-T8, highly suspicious for an acute spinal cord infarction (focal cord swelling and "pencil-like" hyperintensities on T2-weighted images).

**Figure 2:**
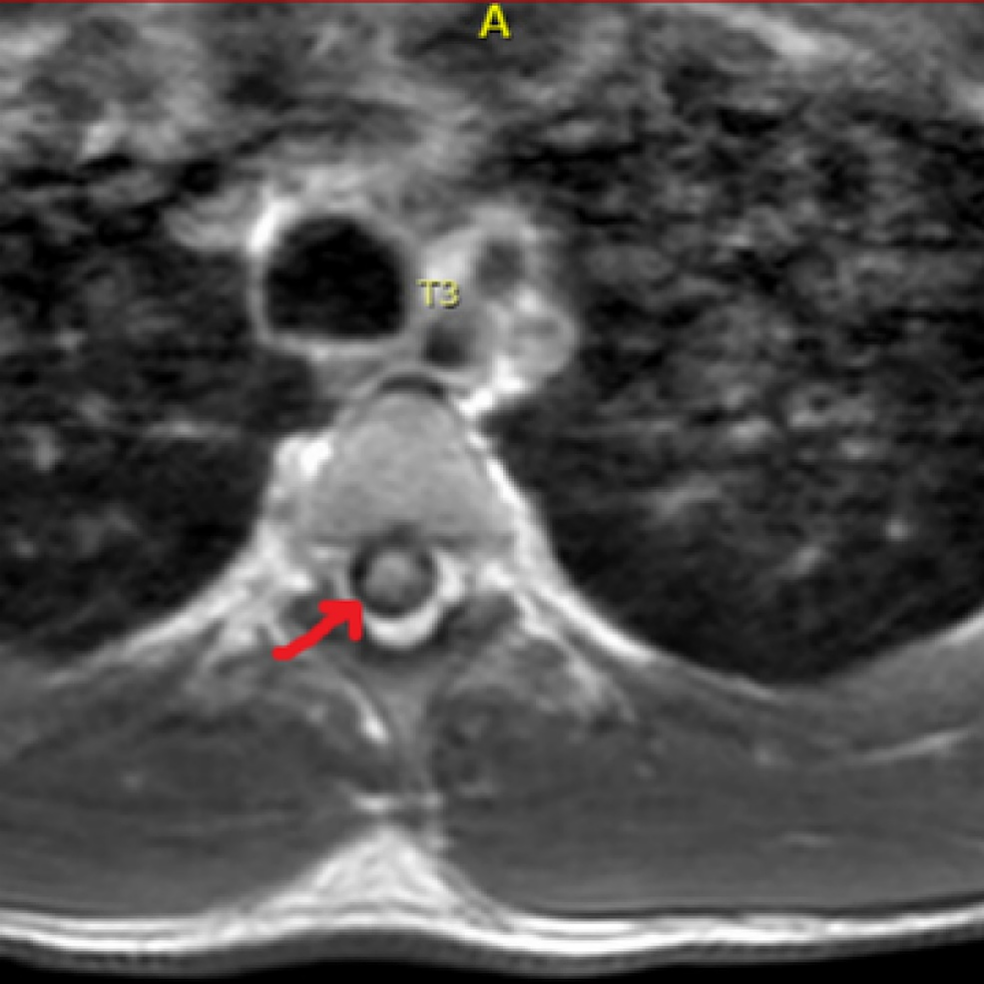
MRI of thoracic spine shows abnormal T2 signaling at the level of T3.

A 23-year-old male with a history of polysubstance abuse presented to the hospital with altered mental status (AMS) and hypoglycemia. He endorsed the use of alcohol, cocaine, and marijuana that day. It was confirmed with a positive toxicology screen. During this hospital admission, his physical examination was notable for paraplegia with no motor abilities from the T6 dermatome and below. Sensation was intact throughout all dermatomes but he was found to have urinary retention, which required a Foley catheter placement. Workup included an abnormal MRI showing T2 signal spanning from T2-T8, raising a high suspicion of a probable acute ischemic SCI (Figures 1, 2). MRI brain was normal, with no changes of demyelination.

A lumbar puncture was performed with extensive serum and cerebrospinal fluid (CSF) studies leading to benign results, which ruled out infectious, autoimmune, and demyelinating causes of the patient’s paraplegia. Several hours after admission, the patient began to exhibit the first signs of abnormal bowel function, initially reported as nausea and sensation of fullness, which progressed to the development of severe abdominal distension and hypoactive bowel sounds. An abdominal x-ray showed the presence of ileus without evidence of obstruction and a nasogastric tube (NGT) was placed with intermittent suction. The next day, NGT was removed, and a low dose of metoclopramide was started. A clear liquid diet was initiated, tolerating it well and resulting in an improvement in bowel sounds. After five days of admission, the patient was able to start a soft mechanical diet with the addition of simethicone for relief of abdominal discomfort. Two weeks later with the sporadic use of bisacodyl suppository for constipation, the patient’s abdominal distension and ileus resolved. Unfortunately, the patient’s bladder dysfunction did not resolve and remained limited to self-urine catheterization. 

## Discussion

The variety and severity of symptoms experienced in SCI depend on the level of the medulla spinalis involved and the associated vascular area. Anterior spinal artery syndrome is the usual clinical manifestation in SCI [8]; and it involves the loss of motor function, temperature sensation, and pain sensation with the preservation of vibratory sense and proprioception below the level of the lesion [9]. Several hypotheses attempting to explain the unclear pathophysiology of cocaine-induced spinal cord infarction include vasospasm, vasoconstriction, vasculitis, platelet aggregation causing thrombosis, and ventricular fibrillation predisposing the patient to embolism [10]. 

Another manifestation of SCI is autonomic dysfunction, causing symptoms that include sexual, bowel, and bladder dysfunction. Neurogenic bowel dysfunction (NBD) can be described as a loss of bowel function secondary to congenital malformation, neurological disorders, or nerve lesions. Overproduction of nitric oxide from the increased regulation of nitric oxide synthase may contribute to the pathophysiology of NBD secondary to spinal cord injury [11]. Frequent symptoms experienced in NBD are fecal incontinence and constipation but abdominal pain and bloating can also be seen. Those symptoms have an overwhelming impact on a patient’s functionality and are often rated as more debilitating than the impact on motor dysfunction [12]. Other factors such as prolonged immobilization and certain medications like opioids, spasmolytics, and antibiotics can contribute to and exacerbate symptoms [13]. 

Liu et al. demonstrated that injury at higher levels of the spinal cord can increase the severity of NBD symptoms [14]. If the lesion is above the conus medullaris (CM), the patient will manifest with constipation and stool retention secondary to hypertonic colon and anal sphincter. If the lesion is at the level or below the CM, the patient will experience constipation along with fecal incontinence, secondary to slow bowel peristalsis and loss of the external anal sphincter tone respectively [7]. 

The management of NBD in patients with SCI requires the assessment of prior and current bowel patterns to individualize the best treatment options. Initially, conservative approaches are applied to optimize the bowel regimen, which includes lifestyle modifications and routine bowel care with scheduled times for defecation, and diet [15]. Adequate fiber intake is recommended, as a high fiber diet can improve the consistency of stools in rapid bowel transit but can produce bloating in slow bowel transit. The amount of fiber intake can be decreased to improve tolerance. The most important diet strategy is a regular eating pattern without skipped meals and optimized fluid intake with consideration for bladder limitations [16]. 

Also, several techniques help with bowel movement depending on the patient’s functionality and include abdominal massage, digital rectal stimulation, Valsalva maneuverer, anal tampons, suppositories, and enemas [17]. Bisacodyl and glycerin suppositories are used to help with stool evacuation; the polyethylene glycol version of bisacodyl is more effective when compared with the vegetable oil-based version [18]. The prokinetic prucalopride has been shown to cause a significant improvement in bowel function and reduction in severity of symptoms in patients with chronic constipation secondary to SCI [19]. Metoclopramide is another prokinetic drug that stimulates the muscles of the gastrointestinal tract and has been used to treat ileus secondary to acute SCI [20].

## Conclusions

We are reporting a rare case of cocaine-induced spinal cord infarction with transient bowel dysfunction symptoms. Clinicians should maintain a high index of suspicion for cocaine use as a cause of nontraumatic spinal cord infarction and be aware of its challenging management due to neurological sequela including the loss of mobility and neurogenic bowel dysfunction. Fecal incontinence and constipation as the result of this dysfunction have an enormous impact on a patient's functional capacity. This overwhelming impact can be experienced as more debilitating than paraplegia or urinary retention in some patients. Multidisciplinary care for those patients can improve their quality of life and decrease health care costs.
